# Antimicrobial Activity and Mechanism of Action of *Dracocephalum moldavica L*. Extracts Against Clinical Isolates of *Staphylococcus aureus*

**DOI:** 10.3389/fmicb.2019.01249

**Published:** 2019-06-06

**Authors:** Hui Yu, Min Liu, Yun Liu, Lei Qin, Min Jin, Zhanli Wang

**Affiliations:** ^1^ The Second Affiliated Hospital, Baotou Medical College, Baotou, China; ^2^ School of Public Health, Baotou Medical College, Baotou, China

**Keywords:** medicinal plant, antimicrobial, cytotoxicity, proteomics, differentially expressed protein, biofilm

## Abstract

**Background:** *Dracocephalum moldavica L.* is a popular traditional medicine used by many countries, which has a wide range of pharmacological effects. The aim of this work was to investigate the antimicrobial effects of *D. moldavica L.* extracts against clinical isolates of *Staphylococcus aureus*. Our results demonstrated that the minimal inhibitory concentration (MIC) for 50 and 90% of *S. aureus* isolates (MIC_50_ and MIC_90_) of the ethyl acetate (EtOAc) fraction from *D. moldavica L*. ethanol extract were 780 and 1,065 μg/ml, respectively. We further observed that the EtOAc fraction disrupted 24-h biofilm caused cell membrane damage and irregular cell shape. Additionally, the EtOAc fraction showed slight to moderate toxic effects on human epidermal keratinocyte (HaCaT) cells. Moreover, the results of the differential proteome revealed that 231 proteins were upregulated, while 61 proteins were downregulated in *S. aureus* after treatment with the EtOAc fraction. The differentially expressed proteins were functionally categorized by the Gene Ontology (GO) enrichment and Kyoto Encyclopedia of Genes and Genomes (KEGG) pathway. These proteins contribute to membrane damage, inhibition of biofilm formation, and changes in energy metabolism. Thus, the EtOAc fraction of *D. moldavica L.* ethanol extract, as a natural product, has the potential to be used as an antimicrobial agent to control the clinical isolates of *S. aureus*.

## Introduction

*Staphylococcus aureus* is a serious human pathogen known to cause numerous bacterial infections at the level of the bloodstream, lower respiratory tract, and skin and soft tissue (Sakr et al., 2018). Penicillin was initially highly effective for treatment of *S. aureus* infections. However, the widespread use of the penicillin led to the emergence of penicillin-resistant *S. aureus* (PRSA) (Chen and Huang, 2014). With the release of beta-lactamase-resistant penicillins such as methicillin and oxacillin in the 1970s, methicillin-resistant *S. aureus* (MRSA) emerged and became an important cause of infectious diseases acquired in hospitals and communities worldwide (McDonald et al., 1981). Since this situation exists, it is necessary to fight against MRSA. Moreover, many recent studies showed that MRSA spread along the food chain, indicating urgent need for control programs to avoid food transmission (Doulgeraki et al., 2017; Oniciuc et al., 2017; Li et al., 2018). However, the treatment of MRSA infections is complicated due to its multi-drug resistance.

Traditional antibiotics are gradually loosing their efficacy against many bacterial pathogens due to fast development of antibiotic resistance in microorganisms. Glycopeptides and several newer classes of antibiotics have proven to have activity against MRSA, including linezolid of the oxazolidinone class and daptomycin of the lipopeptide class (Kali, 2015). However, the drugs available for clinical treatment of MRSA are very limited. Therefore, there is an urgent demand for novel antimicrobial agents able to replace the antimicrobial activity of old antibiotics. Several studies have shown that extracts from plant species may be active against multi-drug resistant bacteria, including MRSA (Martin and Ernst, 2003).

*Dracocephalum moldavica L.* is a medicinal plant used by many countries, which has a wide range of pharmacological effects, including anti-inflammatory, antioxidative, and cardioprotective effects (Jiang et al., 2014). *D. moldavica L*. has been reported to contain steroid, flavonoids, glycoside, saponins, tannins, and phenolic compounds (Zhang et al., 2018). The aim of this work was to investigate the antimicrobial and cytotoxic effects of *D. moldavica L.* extracts. Moreover, the antimicrobial mechanism was also investigated.

## Materials and Methods

### Plant Material and Extracts Preparation

*D. moldavica L.* was collected in the county of Tongliao (latitude 43°39′ north and longitude 122°14′ east), Inner Mongolia, China. The plant material was air dried in shade at room temperature for 7–14 days and ground into powder using an electric grinder (Da Xiang, China). Then, the powdered material (50 g) was subjected to extraction twice with 500 ml of 65% ethanol at a temperature of 60°C for 120 min. The supernatants were then filtered, gathered, and concentrated under vacuum in a rotary evaporator (Yarong RE-2000A, China) to remove ethanol. Finally, the ethanol extract was subjected to extraction with petroleum ether (petrol), dichloromethane (CH_2_Cl_2_), ethyl acetate (EtOAc), and n-butyl alcohol (n-BuOH) using separating funnels. The resultant fractions were evaporated, dried, and stored at −20°C for further experiments.

### High-Performance Liquid Chromatography (HPLC) Analysis

The separation was carried out on a HPLC system (Shimadzu LC-20AT) with a diode array detector (DAD) equipped with Inertsil ODS-SP column (250 × 4.6 mm, 5 μm particle size). Mobile phase A was acetonitrile, and mobile phase B were ultrapure water and acetic acid (999/1, v/v). Gradient conditions were as follows: 10–20 min, linear gradient 70–55% A in B; 20–21 min, linear gradient 55–70% A in B; 21–31 min, isocratic 70–70% A in B. The injection volume was 10 μl. The flow rate was 1.0 ml/min. For qualitative analysis, the fractions were evaluated with the standards (tallianine, rosmarinic acid, luteolin, apigenin, and diosmetin) as the references.

### Bacterial Strains and Antibiotic Susceptibility

The following Gram-positive, Gram-negative bacteria, and fungi strains from the Second Affiliated Hospital of Baotou Medical College were employed: *S. aureus* (120 isolates), *Staphylococcus epidermidis* (6 isolates), *Staphylococcus haemolyticus* (5 isolates), *Enterococcus faecalis* (7 isolates), *Enterococcus faecium* (7 isolates), *Klebsiella pneumonia* (5 isolates), *Escherichia coli*, (10 isolates), *Acinetobacter baumannii* (6 isolates), *Pseudomonas aeruginosa* (9 isolates), *Saccharomyces albicans* (7 isolates), *Candida glabrata* (5 isolates), *Candida krusei* (5 isolates), and *Candida parapsilosis* (3 isolates). *S. aureus* isolates comprised of 23 MRSA and 97 methicillin-sensitive *S. aureus* (MSSA). The clinical isolates used in this study were maintained at −80°C in a freezer. Prior to assay, bacteria and fungi strains were grown overnight at 37 and 35°C, respectively. The isolates were tested for antibiotic susceptibility using BD Phoenix 100 automated system and the conventional Kirby-Bauer agar diffusion disk method as recommended by the Clinical and Laboratory Standards Institute (Clinical and Laboratory Standards Institute, 2018).

### Evaluation of the Antimicrobial Activity

Disk diffusion assays were performed in triplicate for *D. moldavica L.* extracts. Antibacterial and antifungal activities were determined on Mueller Hinton agar and Sabouraud dextrose agar (Oxoid, UK), respectively. Four 6-mm-diameter disks were placed onto each agar plate and then injected with several dilutions of *D. moldavica L*. extracts (0, 50, 100, and 200 μg/ml) with a volume of 10 μl individually. The plates were incubated at temperatures of 37°C for bacteria for 24 h and 35°C for fungi for 24 h. Subsequently, the diameter of inhibition zone surrounding the disk (including the disk) was measured. *S. aureus* ATCC 25923 was used as a quality control strain. The antibacterial activity of EtOAc fraction of *D. moldavica L.* ethanol extract was further evaluated by minimal inhibitory concentration (MIC). A 2-fold broth-dilution method was utilized to assess MIC. The bacterial population was exposed to several dilutions of EtOAc fraction and incubated at 37°C for 16 h. An ELISA reader was used to detect MIC at 595 nm. The MIC of EtOAc fraction which inhibits 50 and 90% of the isolates was denoted MIC_50_ and MIC_90_, respectively.

### Biofilm Formation Assay

*S. aureus* biofilms were allowed to form in the media (TSB + 1% glucose) at 37°C for 24 h in a 6-well plate. After removal of the medium, the biofilms were washed five times with PBS. EtOAc fraction of *D. moldavica L.* ethanol extract was then added to the mature biofilms in the media (TSB + 1% glucose). The plates were incubated at 37°C during 24 h, and then the medium and non-adherent cells were removed by washing with sterile PBS. The effect of EtOAc fraction on biofilm formation was determined using a standard 3-(4,5-dimethyl-2-thiazolyl)-2, 5-diphenyl-2H-tetrazolium bromide (MTT) reduction assay as described previously by Jia et al. (Jia et al., 2011). Ciprofloxacin (50 μg/ml) was used as positive control.

### Scanning Electron Microscopy

In order to observe bacterial morphology under treatment of EtOAc fraction of *D. moldavica L.* ethanol extract *via* a scanning electron microscopy analysis, *S. aureus* was cultured in 60 ml of Mueller-Hinton Broth (MHB) overnight with or without EtOAc fraction treatment. Cells were harvested by centrifugation and washed three times with 0.1 M sodium phosphate buffer solution. Aliquots (20 μl) of *S. aureus* suspensions were deposited onto micro cover glass slides for 30 min and then were immersed into 50 ml of a fixative solution containing 2.5% glutaraldehyde (Electron Microscopy Sciences, Hatfield, PA, USA) for 30 min. The rest of the procedure was as described previously by Suo et al. (Suo et al., 2018). Morphological alterations of the bacteria were observed and photographed using a Quanta 200 FEG environmental scanning electron microscope (FEI Co., Inc., Hillsboro, OR, USA).

### Detection of Cell Membrane Damage

The cell membrane damage caused by EtOAc fraction of *D. moldavica L.* ethanol extract was determined using a phosphorous leakage method. In briefly, bacteria were harvested at the end of logarithmic phase by centrifugation and resuspended in the 5 ml HEPES-Na buffer (5 mM, pH 7.0). Bacterial suspensions were added with 5 μl of the extract dissolved in dimethyl sulfoxide (DMSO) at final concentration of 300 μg/ml. DMSO was used as negative control. The samples were then incubated at 37°C for 1, 2, 3, 5, 10, 15, 20, 25, 30 h, respectively. Then the total phosphate was determined as described previously by Luo et al. (Luo et al., 2015).

### Cytotoxicity Evaluation

The *in vitro* cytotoxicity of EtOAc fraction of *D. moldavica L.* ethanol extract was investigated using human epidermal keratinocyte (HaCaT) cell line as described previously by Wadhwa et al. (Wadhwa et al., 2019). HaCaT cells were cultivated in Dulbecco’s modified Eagle’s medium (DMEM) medium supplemented in 10% fetal bovine serum (FBS, Thermo Fisher Scientific, MA, USA), streptomycin (100 μg/ml, Thermo Fisher Scientific, MA, USA), and penicillin (100 IU/ml, AppliChem GmbH, Darmstadt, Germany) in a humidified atmosphere containing 5% CO_2_ at 37°C. EtOAc fraction was dissolved in distilled DMSO and DMEM supplemented with 2% FBS. From the stock solution (1 mg/ml), serial two-fold dilutions (0–100 μg/ml) were prepared for analysis. Cells were seeded in a 96-well plate at 1 × 10^5^ cells per well. After 24 h, cells were treated with serial dilutions of EtOAc fraction and incubated for 24 h. After incubation, 0.5 mg/ml of MTT was added to each well for 2 h. Absorbance was measured at 590 nm using a microplate reader.

### Peptide Separation and Identification by Non-Labeled HPLC-MS

Cells were harvested by centrifugation of 5 ml of bacterial culture. After triple washing with PBS, the bacterial pellet was resuspended in lysis buffer (2% SDS, 7 M urea, 1 × Protease Inhibitor Cocktail), followed by sonication on ice for 30 min. Then, samples were centrifuged at 15,000 rpm for 15 min at 4°C. The supernatant was used for the measurement of protein concentration with a BCA Assay Kit (Sigma-Aldrich, St. Louis, MO, USA). The sample was then digested to produce the final trypsinized peptide sample as described previously by Suo et al. (Suo et al., 2018). The fraction was separated by Easy-nLC 1000 system (Thermo Fisher Scientific, MA, USA) and analyzed by Q-Exactive mass spectrometer (Thermo Fisher Scientific, MA, USA) equipped with an online nano-electrospray ion source. Peptide sample was loaded onto the trap column (Thermo Scientific Acclaim PepMap C18, 100 μm × 2 cm), with a flow of 10 μl/min for 3 min and subsequently separated on the analytical column (Acclaim PepMap C18, 75 μm × 15 cm) with a linear gradient, from 3 to 32% D in 120 min. The column flow rate was maintained at 300 nl/min. The electrospray voltage of 2 kV versus the inlet of the mass spectrometer was used. The mass spectrometer was run under data dependent acquisition mode and automatically switched under MS and MS/MS mode. MS spectra were acquired across a mass range of 350–1550 m/z. The dynamic exclusion time was set as 20 s.

### Database Search, Protein Identification, and Expression Quantification

Spectrograms were used for protein identification using PEAKS Studio version 8.0 (Bioinfor Inc. CA) by querying the UniProt database, which contains 9781 protein sequences. Peptides were filtered by 0.01% false discovery rate (FDR). PEAKS Q was used for peptide and protein abundance calculation. Normalization was performed on averaging the abundance of all peptides. Different expressed proteins were filtered if their log2 fold change >2.0 fold and statistical *p* < 0.01. The Gene Ontology (GO) analysis was performed using BLAST2GO version 4. Whole protein sequence database was analyzed by BlastP using whole database and mapped, annotated with gene ontology database. Statistically altered functions of different expressed proteins were calculated by Fisher’s exact test in BLAST2GO. Kyoto Encyclopedia of Genes and Genomes (KEGG) pathway analysis was processed by KOBAS^1^. Pathways with *p* < 0.05 were recognized as significant changed.

### Statistical Analysis

Statistical analysis was performed with SPSS20.0 software. Analysis of variance (ANOVA) and Tukey multiple range test were used to test the difference. Values of *p* < 0.05 were considered statistically significant.

## Results

### Antimicrobial Activity of *D. moldavica L*. Extracts

Four types of fractions from *D. moldavica L*. ethanol extract were obtained: petrol, CH_2_Cl_2_, EtOAc, and n-BuOH. The antimicrobial activities of the fractions of *D. moldavica L*. were evaluated by the agar diffusion test. In this test, disks containing increasing extract concentrations are placed on an agar plate where organisms have been placed. The EtOAc fraction showed significant antibacterial activity against five Gram-positive bacteria (*S. aureus*, *S. epidermidis*, *S. haemolyticus*, *E. faecalis*, and *E. faecium*) in a dose-dependent manner. However, no antimicrobial activity against Gram-negative bacteria and fungi tested was seen (concentration up to 250 mg/ml). Additionally, no antimicrobial activities against both Gram-positive and Gram-negative bacteria and fungi were observed for petrol, CH_2_Cl_2_, and n-BuOH fractions of *D. moldavica L.* (concentration up to 250 mg/ml). Moreover, our results found that the MIC values of the EtOAc fraction for *S. aureus* isolates ranged from 390 to 1,560 μg/ml with an MIC_50_ of 780 μg/ml and an MIC_90_ of 1,065 μg/ml.

### Phytochemical Analysis of EtOAc Fraction of *D. moldavica L*. Ethanol Extract

**Figure 1:**
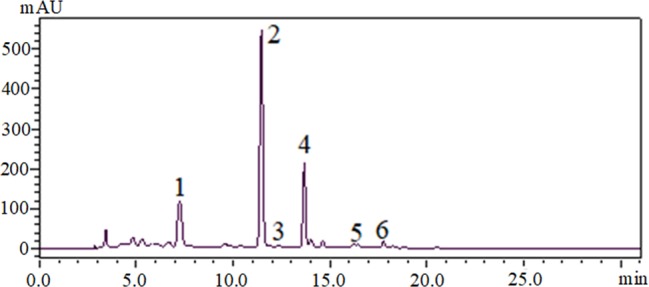
HPLC-DAD chromatograms of EtOAc fraction of *D. moldavica L*. ethanol extract, visualized at 330 nm. 1, rosmarinic acid; 2, tilianin; 3, luteolin; 4, an unknown compound; 5, apigenin; 6, disometin.

Through HPLC analysis of the EtOAc fraction, several compounds were identified. The majority compounds found in the EtOAc fraction were rosmarinic acid (peak 1), tilianin (peak 2), and an unknown compound (peak 4) (Figure 1). The chromatograms also showed that luteolin (peak 3), apigenin (peak 5), and disometin (peak 6) were found in the EtOAc fraction (Figure 1).

### Effect of EtOAc Fraction of *D. moldavica L*. Ethanol Extract on Membrane Integrity

**Figure 2:**
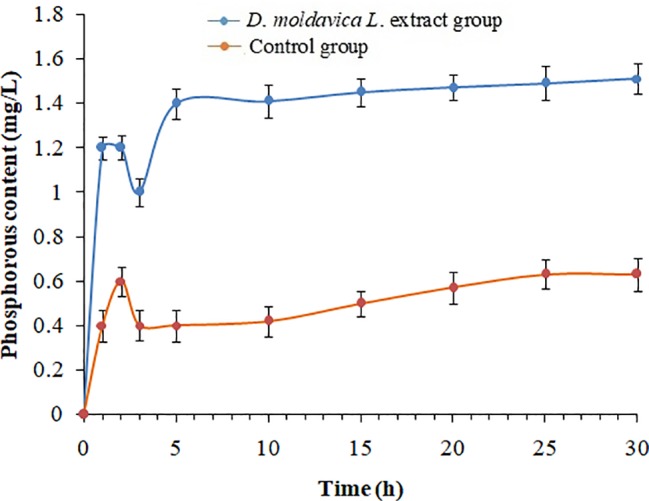
Phosphorous leakage of *S. aureus* treated with and without EtOAc fraction of *D. moldavica L*. ethanol extract at different time.

The cell membrane integrity of MRSA isolates was measured by UV-absorbing release materials (Figure 2). Compared with control group, the release of phosphate ions from EtOAc fraction group was remarkably increased (*p* < 0.05). The significant difference in increase of phosphate ion leakage was observed within 3 h, and the levels of phosphate ion leakage were stable during treatment of EtOAc fraction of *D. moldavica L*. ethanol extract for 10 h.

### Effect of EtOAc Fraction of *D. moldavica L*. Ethanol Extract on Bacterial Morphology

**Figure 3:**
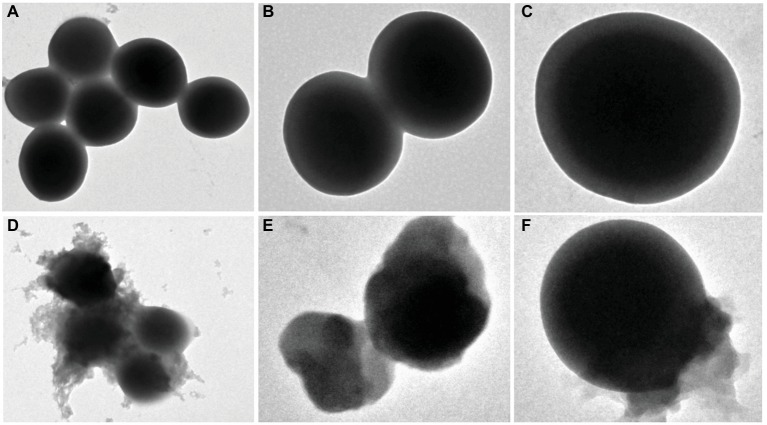
Scanning electron microscopy analysis of *S. aureus* treated with and without EtOAc fraction of *D. moldavica L*. ethanol extract. Images **(A)** and **(D)** were observed at an instrumental magnification of 10,000×; images **(B)** and **(E)** at an instrumental magnification of 20,000×; images **(C)** and **(F)** at an instrumental magnification of 60,000×. **(A–C)** *D. moldavica L*. extract group. **(D–F)** Control group.

Changes in the bacterial morphology of MRSA isolates after exposure to EtOAc fraction were observed by scanning electron microscopy analysis. The untreated bacteria were used as the control. As shown in Figure 3, the untreated bacteria displayed a smooth and intact surface with typical morphology. After incubation with EtOAc fraction of *D. moldavica L*. ethanol extract, bacteria showed the leakage of bacterial content, leading to dramatic changes in morphology. Clearly, these cells were smaller in size than controls. Lysed cells and debris were also observed.

### Effect of EtOAc Fraction of *D. moldavica L*. Ethanol Extract on Biofilm Formation

**Figure 4:**
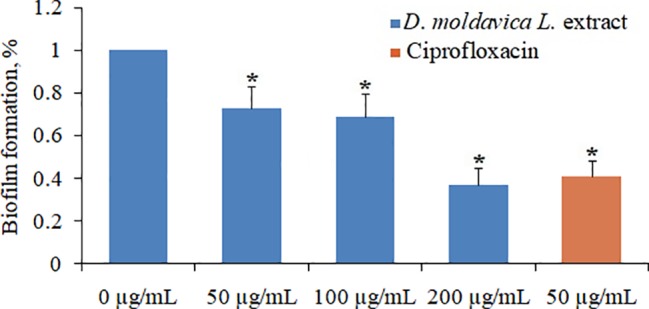
Effect of EtOAc fraction of *D. moldavica L*. ethanol extract on biofilm formation of *S. aureus* at concentrations of 0–200μg/ml. ^*^D. moldavica L. extract-treated group vs. untreated group, *p* < 0.05 were considered significant.

EtOAc fraction of *D. moldavica L*. ethanol extract exhibited pronounced dose-dependent inhibitory effects on biofilm formation of tested MRSA strain (Figure 4). EtOAc fraction at doses of 50, 100, and 200 μg/ml was able to inhibit formation of *S. aureus* 24-h biofilms by 27, 31, and 63%, respectively, as compared to the untreated control.

### Effect of EtOAc Fraction of *D. moldavica L*. Ethanol Extract on Cytotoxicity

The cytotoxicity of EtOAc fraction was carried out using MTT assay. The results of HaCaT cell line treated with different concentrations of EtOAc fraction are presented in Table 1. The EtOAc fraction at doses of 20, 40, 60, 80, and 100 μg/ml was able to inhibit proliferation of HaCaT cell line by (6.03 ± 0.39), (9.17 ± 0.41), (17.79 ± 0.97), (25.13 ± 0.73), and (33.27 ± 0.71)%, respectively.

**Table 1:** *In vitro* cytotoxicity of *D. moldavica L*. extract.

Concentration (μg/ml)	% Inhibition
0	0.00
20	6.03 ± 0.39
40	9.17 ± 0.41
60	17.79 ± 0.97
80	25.13 ± 0.73
100	33.27 ± 0.71

### Effect of EtOAc Fraction of *D. moldavica L*. Ethanol Extract on Protein Expression

**Figure 5:**
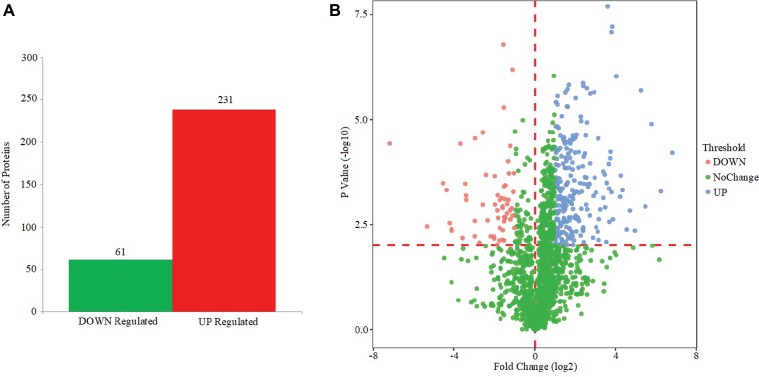
Effect of EtOAc fraction of *D. moldavica L*. ethanol extract on protein expression. **(A)** Identification of differentially expressed proteins in *S. aureus* following EtOAc fraction treatment. **(B)** The volcano of differentially expressed proteins.

Based on the MS analysis of each sample, changes in the protein profile were analyzed. The present study showed that 292 proteins exhibited a difference (fold changes ≥ 2) with a FDR of less than 0.01% (Figure 5A). Among 292 differentially expressed proteins, 231 proteins were significantly upregulated, while 61 proteins were significantly downregulated. Figure 5B shows the volcano of differentially expressed proteins. The top 20 upregulated proteins included undecaprenyl-diphosphatase, delta-hemolysin, and choline dehydrogenase (Table 2). The top 20 downregulated proteins were listed in Table 3, including copper chaperone copZ, antiholin-like protein lrgB, and site-specific tyrosine recombinase.

**Table 2:** Top 20 increased expressed proteins in *D. moldavica L*. extract treatment group compared to control.

Accession	Description	Gene name	Fold change	*p*
Q2G0B4	Undecaprenyl-diphosphatase	UppP	75.71	0.000515
G5JKT9	Delta-hemolysin	Hld	26.06	0.001483
Q2FV11	Choline dehydrogenase	BetA	23.35	0.004140
Q2G093	Glycerol phosphate lipoteichoic acid synthase	LtaS1	18.68	0.000693
Q932F7	MSCRAMM family adhesin	SdrE	14.6	0.002377
Q2FZZ2	Methionine ABC transporter ATP-binding	MetN2	11.26	0.001239
Q2FWG4	Membrane insertion	OxaA	10.54	0.000175
Q2G0Q9	Chaperonin (heat shock 33)	HslO	10.52	0.000740
Q2G273	Urease accessory	UreG	9.51	0.001393
Q2FYG5	Ubiquinone menaquinone biosynthesis methyltransferase	UbiE	7.32	0.006516
Q2FXN3	Citrate synthase (si)	CitZ	6.57	0.001923
Q2FY68	Pyrroline-5-carboxylate reductase	ProC	6.5	0.000341
G5JHQ8	Nucleoside diphosphate kinase	Ndk	5.92	0.000352
Q2FWB7	Non-specific DNA-binding Dps Iron-binding ferritin-like antioxidant Ferroxidase	Dps1	5.81	0.000240
Q9EZ11	Dihydrodipicolinate reductase	DapB	5.72	0.00
Q2FVL3	Cystine transport system permease	TcyB	5.57	0.000234
Q2FW99	PTS system mannitol-specific transporter subunit IIB	MtlA	4.88	0.00
Q2FWY4	DASS family divalent anion:sodium (Na+) symporter	SdcS	4.68	0.000325
Q2G0Z5	FMN reductase	NfrA	4.38	0.002173
Q2FYG9	Chorismate synthase	AroC	3.62	0.000482

**Table 3:** Top 20 decreased expressed proteins in *D. moldavica L*. extract treatment group compared to control.

Accession	Description	Gene name	Fold change	*p*
Q2FV63	Copper chaperone CopZ	CopZ	−40.38	0.003516
P60643	Antiholin-like protein LrgB	LrgB	−17.51	0.004055
Q2FZ30	Site-specific tyrosine recombinase	XerC	−11.92	0.006561
Q931E9	Surface SAV2496 SAV2497	SasG	−10.49	0.000826
Q2FXV6	tRNA (5-methylaminomethyl-2-thiouridylate)- methyltransferase	TrmU	−7.86	0.002588
Q2G1D7	Pyruvate formate-lyase activating enzyme	PflA	−4.09	0.006067
Q2FX12	Low molecular weight tyrosine phosphatase	YfkJ	−3.99	0.000223
Q2FVM4	Respiratory nitrate reductase gamma chain	NarI	−3.66	0.009594
Q2FY19	Zinc uptake regulation ZUR	Zur	−3.63	0.001459
Q2FVM2	Respiratory nitrate reductase beta chain	NarH	−3.60	0.000643
Q2G2U1	Sensor histidine kinase	SaeS	−3.53	0.002208
Q2G2M7	Serine acetyltransferase	CysE	−3.39	0.007534
Q2G0B7	Lysine decarboxylase family	YvdD	−3.12	0.000743
Q2FWD8	50S ribosomal L31 type B	RpmE	−3.01	0.001282
Q2G1D8	Formate acetyltransferase	PflB	−2.88	0.00
Q2G0Q0	Pyridoxine biosynthesis amidotransferase	PdxT	−2.73	0.000367
Q2FYK6	Dihydrofolate reductase	FolA	−2.47	0.00
Q2FVL9	Nitrite reductase [NAD(P)H] small subunit	NirD	−2.42	0.000192
Q2FYF1	Cell surface elastin binding	EbpS	−2.32	0.001455
Q2FVZ4	Glycine glycyltransferase	FemX	−2.30	0.002388

### Functional Categorization of Differentially Expressed Proteins

**Figure 6:**
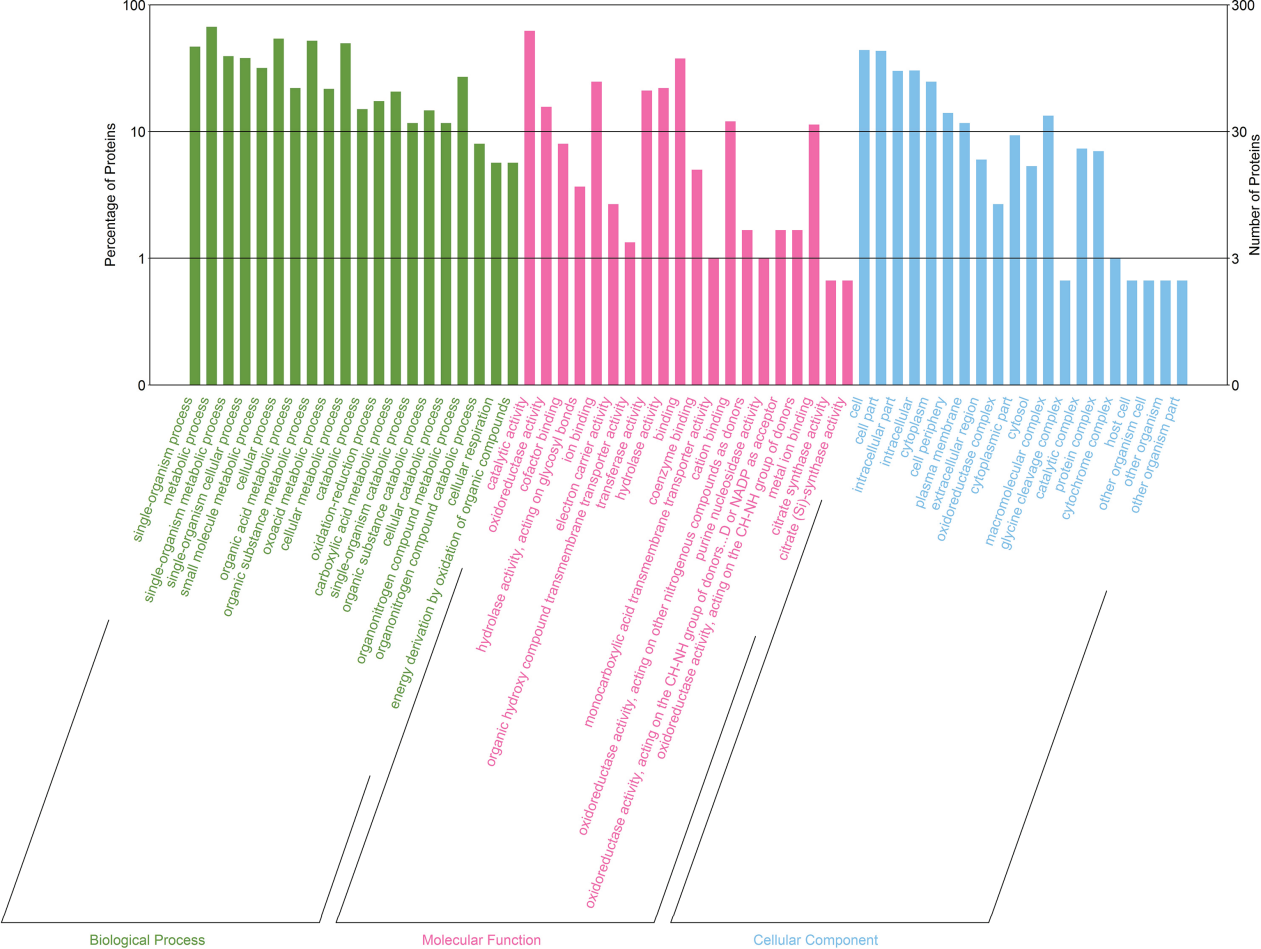
GO enrichment analysis of differentially expressed proteins between *D. moldavica L*. extract-treated cells and control cells of *S. aureus*. The x-axis represents top 20 GO terms of three categories (biological processes, molecular functions, and cellular components). The y-axis represents the number and percentage of proteins.

Next, we classified the upregulated and downregulated proteins based on the relative categories annotated by the GO method (Figure 6). Among the differentially expressed proteins classified as being involved in biological process, 140, 201, and 118 proteins were classified into the categories of single-organism process (GO: 0044699; *p* = 3.98E−18), metabolic process (GO: 0008152; *p* = 2.29E−16), and single-organism metabolic process (GO: 0044710; *p* = 3.02E−16), respectively. For molecular function, 187, 47, and 24 proteins were classified into the categories of catalytic activity (GO: 0003824; *p* = 1.18E−18), oxidoreductase activity (GO: 0016491; *p* = 1.46E−09), and cofactor binding (GO: 0048037; *p* = 1.80E−06), respectively, which were catalytic activity and binding related. Moreover, for cellular component, a majority were intracellular related.

### KEGG Pathway Analysis of Differentially Expressed Proteins

**Figure 7:**
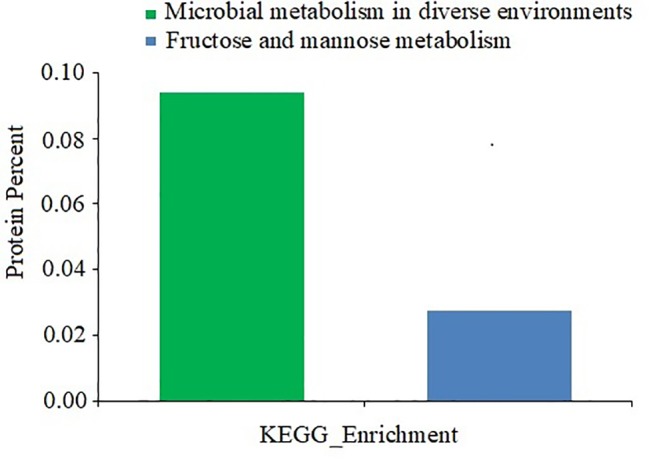
KEGG pathway analysis of differentially expressed proteins between *D. moldavica L*. extract-treated cells and control cells of *S. aureus*. The x-axis represents different KEGG categories. The y-axis represents the number of proteins.

The functions of the differentially expressed proteins were further analyzed by the KEGG pathway annotation method and 39 pathways were obtained (Figure 7). Only two enriched pathways had a *p* of less than 0.05: microbial metabolism in diverse environments (*p* = 0.029), and fructose and mannose metabolism (*p* = 0.031).

## Discussion

MRSA has become an increasingly important pathogen in hospitals and the community (Campanile et al., 2009). Current antibiotic treatment of MRSA infection has become limited due to the emergence of multidrug-resistant isolates. *D. moldavica L*. is one of the most widely used medicinal plants. To date, however, it is still not known whether *D. moldavica L*. has antimicrobial activity. In the study, we investigated the antimicrobial effects of *D. moldavica L*. extracts.

The preliminary antibacterial activity results showed that the EtOAc fraction of *D. moldavica L*. ethanol extract exhibited remarkable antibacterial activity against MRSA isolates with an MIC_50_ of 780 μg/ml and an MIC_90_ of 1,065 μg/ml. Additionally, agar diffusion test revealed that this fraction exhibited significant antibacterial activity in a dose-dependent manner, indicating that the EtOAc fraction of *D. moldavica L*. showed promising capacity as new phytotherapeutic candidate against MRSA.

In this study, we showed that tilianin, rosmarinic acid, and unknown compound were the main compounds in the EtOAc fraction of ethanol extract of *D. moldavica L*. Therefore, the antibacterial properties against microorganisms of EtOAc fraction can be explained by the antimicrobial activity of its phytoconstituents, such as tannins and rosmarinic acid, which have shown to have effective antibacterial properties (Bigos et al., 2012; Ekambaram et al., 2016).

Changes in the cell morphology of MRSA isolates were detected by scanning electron microscopy analysis. Results showed that MRSA isolates treated with EtOAc fraction exhibited irregular cell shape and a high degree of cell lysis. These results can be explained by assuming that the EtOAc fraction may damage cell membrane. Moreover, the EtOAc fraction promotes the phosphate ion leakage of MRSA isolates, implicating that this fraction can react with bacterial membrane and increase the membrane permeability. In fact, as already mentioned, extracts of some herbal medicines can damage the phospholipids bilayer in the plasma membrane resulting in leaked cytoplasmic material of the bacteria (Tsuchiya et al., 1996; Maia et al., 2011). Our results seem consistent with previous findings.

We further evaluated the cytotoxicity of the EtOAc fraction against the cell line HaCaT. The cytotoxicity assay showed the cell proliferation inhibition rate of (33.27 ± 0.71)% for the EtOAc fraction at high concentration (100μg/ml). Our results indicated that the treatment of HaCaT cells with EtOAc fraction did not result in significant cytotoxic effect. We could not find previously published studies on the cytotoxicity of these extracts.

To elucidate the possible molecular mechanism of antibacterial action, we used non-labeled HPLC-MS quantitative proteomic analysis to compare the proteomic pattern of MRSA isolates after exposure to the EtOAc fraction. Several proteins were upregulated that might play a role in the antimicrobial activity of the EtOAc fraction. These proteins include undecaprenyl-diphosphatase, delta-hemolysin, and choline dehydrogenase. Undecaprenyl-diphosphatase was involved in peptidoglycan biosynthetic and regulation of cell shape (Wang et al., 2017). Thus, under the burden of severe environment, it may involve in the protective response to external stress to maintain the cell shape. Similarly, glycine betaine is necessary for the survival of bacteria in diverse environment (Yilmaz and Bülow, 2002). Choline dehydrogenase oxidizes choline to betaine aldehyde and then further on to glycine betaine. Upregulated gene expression of glycine betaine biosynthesis-related enzymes induced by stress is clearly observed (Xu et al., 2018). Besides the previously reported hemolytic activity, delta-hemolysin is a novel endogenous molecule that inhibits bacterial self-motility due to its detergent effects, which interfere with the hydrophobic nature of *S. aureus* cell surfaces (Omae et al., 2012). Thus, delta-hemolysin might be a target of the EtOAc fraction of *D. moldavica L*. ethanol extract to damage bacterial cell surfaces. In comparison, several proteins were downregulated, including copper chaperone copZ, antiholin-like protein lrgB, and site-specific tyrosine recombinase. The copZ is a key element of copper homeostasis in bacteria. Recent reports showed that copZ may have an independent role in bacterial biofilm formation and competitiveness through novel virulence-regulatory pathways (Garcia et al., 2016). Coincidentally, lrgB regulates cell envelope and transport related processes (Lama et al., 2012). In *S. aureus*, lrgB was shown to encode holin-antiholin-like protein, which was involved in programmed cell death and biofilm formation. Enhanced expression of genes encoding holin-antiholin-like proteins may be one of the main reasons for early biofilm formation (Chen et al., 2015). Moreover, previous studies demonstrated that members of the tyrosine site-specific recombinase family, conserved in microorganism, mediate additional DNA inversions of the genome, which regulates the synthesis of multiple surface proteins (Weinacht et al., 2004). Taken together, those differentially expressed proteins involved in environmental stress response and the regulations of cell surface and biofilm formation.

To understand the gene functions associated with the differentially expressed proteins, the GO enrichment analysis and KEGG pathway analysis were performed. The GO enrichment analysis indicated that the differentially expressed proteins were predominantly metabolism associated. KEGG pathway analysis predicted that the differentially expressed proteins are associated with microbial metabolism in diverse environments as well as fructose and mannose metabolism. In fact, adaption to an environmental stress after treatment of the EtOAc fraction of *D. moldavica L*. ethanol extract requires a shift in the expression of proteins involved in specific metabolic pathways. In this study, pyruvate formate-lyase activating enzyme, formate acetyltransferase, and glyoxylate reductase were downregulated. These proteins were involved in the anabolic pathways, such as glyoxylate cycle and pyruvate fermentation. However, citrate synthase, FMN reductase, and lactose phosphotransferase system repressor were upregulated. Therefore, our results indicated that the EtOAc fraction of *D. moldavica L*. ethanol extract treatment enhanced aerobic bacterial metabolism and inhibited anaerobic metabolism.

## Conclusions

We assessed the antimicrobial activity of the EtOAc fraction of *D. moldavica L*. ethanol extract on MRSA strains. No significant cytotoxic effect was detected on HaCaT cell line. We further revealed that this fraction induced the differentially expressed proteins that ultimately lead to downstream consequences resulting in membrane damage, inhibition of biofilm formation and changes in energy metabolism.

## Ethics Statement

Ethical approval was obtained from the Second Affiliated Hospital, Baotou Medical College Research and Ethical Review Committee. Informed written consent was obtained from each participant in the study. Any data generated from the specimens protected the patent privacy, confidentiality and anonymity.

## Author Contributions

HY and ML experimental strategy, performed experiments and analyses. YL designed and performed experiments. LQ and MJ performed experiments, including the analysis of composition of the *D. moldavica* ethylacetate extract. ZW conceived this research and wrote the paper. All authors approved the final version of the manuscript.

### Conflict of Interest Statement

The authors declare that the research was conducted in the absence of any commercial or financial relationships that could be construed as a potential conflict of interest.

## Footnotes

^1^http://kobas.cbi.pku.edu.cn/

## Abbreviations

CH_2_Cl_2_: Dichloromethane
DMEM: Dulbecco’s modified Eagle’s medium
DMSO: Dimethyl sulfoxide
EtOAc: Ethyl acetate
FDR: False discovery rate
GO: Gene Ontology
HaCaT: Human epidermal keratinocyte
HPLC-MS: High-performance liquid chromatography-mass spectrometry
KEGG: Kyoto Encyclopedia of Genes and Genomes
MHB: Mueller-Hinton Broth
MIC: Minimal inhibitory concentration
MRSA: Methicillin-resistant *Staphylococcus aureus*
MSSA: Methicillin-sensitive *Staphylococcus aureus*
MTT: 3-(4,5-dimethyl-2-thiazolyl)-2, 5-diphenyl-2H-tetrazolium bromide
PRSA: Penicillin-resistant *Staphylococcus aureus*.
